# A general model for predicting enzyme functions based on enzymatic reactions

**DOI:** 10.1186/s13321-024-00827-y

**Published:** 2024-03-31

**Authors:** Wenjia Qian, Xiaorui Wang, Yu Kang, Peichen Pan, Tingjun Hou, Chang-Yu Hsieh

**Affiliations:** 1 College of Pharmaceutical Sciences, Zhejiang University, Hangzhou, 310058 Zhejiang China; 2 Dr. Neher’s Biophysics Laboratory for Innovative Drug Discovery, State Key Laboratory of Quality Research in Chinese Medicine, Macau Institute for Applied Research in Medicine and Health, Macau University of Science and Technology, Macao, 999078 China; 3 CarbonSilicon AI Technology Co., Ltd, Hangzhou, 310018 Zhejiang China

## Abstract

Accurate prediction of the enzyme comission (EC) numbers for chemical reactions is essential for the understanding and manipulation of enzyme functions, biocatalytic processes and biosynthetic planning. A number of machine leanring (ML)-based models have been developed to classify enzymatic reactions, showing great advantages over costly and long-winded experimental verifications. However, the prediction accuracy for most available models trained on the records of chemical reactions without specifying the enzymatic catalysts is rather limited. In this study, we introduced BEC-Pred, a BERT-based multiclassification model, for predicting EC numbers associated with reactions. Leveraging transfer learning, our approach achieves precise forecasting across a wide variety of Enzyme Commission (EC) numbers solely through analysis of the SMILES sequences of substrates and products. BEC-Pred model outperformed other sequence and graph-based ML methods, attaining a higher accuracy of 91.6%, surpassing them by 5.5%, and exhibiting superior F1 scores with improvements of 6.6% and 6.0%, respectively. The enhanced performance highlights the potential of BEC-Pred to serve as a reliable foundational tool to accelerate the cutting-edge research in synthetic biology and drug metabolism. Moreover, we discussed a few examples on how BEC-Pred could accurately predict the enzymatic classification for the Novozym 435-induced hydrolysis and lipase efficient catalytic synthesis. We anticipate that BEC-Pred will have a positive impact on the progression of enzymatic research.

## Introduction

Enzymes are macromolecules that are responsible for essential chemical reactions in living organisms and beyond. In fact, they participate in nearly all metabolic activities in a living organism in order to produce energy and carry out other life-critical tasks [1, 2], even including the regulation of exogenous substances, such as drug metabolism [3]. In addition to their indispensable roles in sustaining life, with the rapid development of synthetic biology, enzymes-catalyzed reactions have also been engineered and exploited towards the synthesis of complex molecules that could be highly valuable in biomedical field and other industries. More specifically, existing enzymes can react with newly identified substrates to synthesize new products or newly developed enzymes can be used to catalyze entirely novel chemical reactions to greatly expand the synthesizable regions in the vast chemical space. All these impressive developments are made possible due to the advancement of biocatalytics, cheminformatics, artificial intelligence and other computational approaches, which have become instrumental in desgining novel enzyme-based catalysts and reactions. In short, enzyme engineering has profoundly transformed many aspects of chemistry, biotechnology and medicine.

Although the UniProt database encompasses entries over 36 million distinct enzymes, more than 99% of them lack high-quality annotations for catalytic reactions. Despite the existence of numerous methods and platforms for the exploration of enzyme components, identifying enzymes specific to catalyzing particular reactions remains a formidable challenge. To know what kind of enzymes can catalyze a particular chemical reaction, it is important to accurately identify the enzyme commission (EC) number associated with a reaction. In fact, EC numbers represent not only a hierarchical classification of enzyme-catalyzed chemical reactions but also the identifiers for enzymes or enzyme genes in complete genome analyses. This duality of EC numbers makes it possible to relate biological information (such as enzymatic genes) to chemical reactions in metabolic pathways [4]. EC number is a four-level hierarchy; for instance, 3.1.1.1. The first digit indicates the kind of chemical reactions that an enzyme can catalyze, identified as EC 1 through EC 7. The second and third digits delineate further microscopic details, including the type of chemical bonds, the functional groups, and the cofactors involved in a catalytic reaction. The fourth digit encodes the specificity of substrates, which provide matching criteria to specific enzyme. Therefore, determining the EC number of a particular enzyme has enormous implications for understanding its function. Regrettably, the EC system lacks specified EC numbers for numerous known metabolic reactions, as the publication of complete enzyme properties is a prerequisite for their assignment. In fact, the EC numbers for the majority of enzymes in the secondary metabolism are unlikely to be obtained, since many reaction steps cannot be fully characterized by conventional methods [4]. Given the condition above, it is desirable to develop an automatic EC number assignment system for enzymatic reactions to quickly and accurately identify metabolic reactions and metabolites. Furthermore, this system could also provide insights on annotating protein function, identifying possible catalytic methods for unlabeled reactions and thereby connecting the genomic library for enzymatic genes with the chemical library of metabolic pathways.

Since it is time-consuming, laborious and costly to obtain EC numbers required for enzymatic reactions by conventional experiments, many machine learning (ML) and data-driven approaches have been developed. In numerous current models, enzymes are typically predicted to correspond to their Enzyme Commission (EC) numbers based on various characteristics, including amino acid composition, the presence of functional domains, pseudo-amino acid composition, and similarities in protein sequences [5–12]. In a recent publication, Zhao et al. introduced a machine learning algorithm named CLEAN (Comparative Learning-enabled Enzyme Annotation), designed to predict enzyme functions from amino acid sequence characterizations. This innovative approach is applicable even for enzymes that have hitherto been unexplored or poorly understood [13]. Although some of these models based on protein sequence representation have achieved extremely high prediction accuracy [10], only few ML algorithms tried to study enzymatic function from the perspective of chemical reactions waiting to be catalyzed. The assignment of Enzyme Commission (EC) numbers to enzymatic reactions is an arduous and continual task that necessitates the perpetual generation and refinement of rules by domain experts. Automating the annotation process presents a viable avenue to alleviate the dependence on expert curation. Currently, the datasets available for annotating enzyme-catalyzed reactions are notably limited in size, and exhibit a skewed distribution of enzyme classes. This paucity and disparity present considerable challenges to the effective deployment of data-intensive deep learning techniques, which in turn compromises the predictive accuracy, particularly for less represented enzyme classes. By leveraging the capabilities of deep learning, we can address these issues of data insufficiency and imbalance, enabling the processing of constrained datasets more effectively. Furthermore, the majority of current prediction methodologies remain constrained to models that are trained exclusively on individual molecular entities. These models do not extend to the complexities inherent in reactions, which encompass interactions and transformations between molecules.

In 2018, Cai et al. [3] used ML methods along with reaction fingerprints (as the choice of data featurization) to construct a multi-classification model to predict enzyme reactions catalyzed by hydrolases (EC 3.x.x.x) and redox enzymes (EC 1.x.x.x), which achieved excellent predictive performances. However, the model could only make EC-level 3 predictions for two (out of seven) EC classes, and therefore the application scope of this model is rather restricted. In 2022, Watanabe et al. established 16 extended enzymatic reaction prediction models by employing various ML algorithms, including deep neural networks (DNN) [14]. However, when employing solely substrate and product (SP) information for predictions, their method exhibited reduced accuracy (F1 score = 0.654), and no readily available open-source model could be directly applied. One technical challenge is the prediction or inference of the underlying biological information on enzymes based solely on molecular representations of substrates and products. Despite these challenges, there is a clear and pressing need to develop predictive capabilities that focus on a chemical reaction and determine which enzyme family could catalyze the given reaction. This study aims to address these gaps by leveraging deep learning to process limited datasets more effectively and to extend the prediction methodology to encompass the full spectrum of enzymatic reactions.

Recently, a very popular deep learning (DL) model Transformer and its derivatives have been widely adopted for application in chemistry with very impressive results (especially, in the field of organic chemistry such as the synthesis planning). However, due to data scarcity, Transformer has never been adopted for EC classification, as it is challenging to directly train such a large and complex model based on the limited enzyme reaction data. One way to get around the obstacle of limited data is to exploit additional data in a related domain via transfer learning. Generally, it could be very beneficial when an appropriate dataset is selected for pre-training, as a DL model can capture a broad knowledge base that prepares the model for the downstream tasks. More precisely, in this study, we consider a large dataset comprising generic (but mainly organic) reactions for training a Transformer model. As to be explained later, the model succssefully picks up rules of chemical reactions (highly relevant to enzymatic reactions) in an unsupervised manner [15], and establishes a new state-of-the-art performances on the classification of EC numbers for chemical reactions [3, 4, 14].

Our newly proposed protocol for preparing a learnable representation from Transformer (BERT)-based EC prediction model named BEC-Pred for predicting enzyme classification, holding a noticeable advantage over all previous methods [15, 16]. On this basis, the reaction fingerprints generated by our model allowed us to map enzymatic reactions into chemical space and to visualize (using TMAPs [17]) a complete clustering of these embedded reactions, providing additional insight into the underlying chemistry of these systems. In the present study, we conducted a comparative analysis of our proposed model against other widely used classification approaches for chemical reactions. To this end, we put forth three distinct classification methodologies, namely K-Nearest Neighbor (KNN), Random Forest (RF), and Multi-Layer Perceptron (MLP), all of which were centered on the reaction difference fingerprints. In addition, we developed dedicated KNN and MLP classifiers that were based on drfp fingerprints [18], as well as a Graph Neural Network (GNN) classifier [19] that leveraged reaction graphs. All classification models were trained and assessed on the identical dataset, thus facilitating an equitable and thorough comparison of our model against its counterparts. Ultimately, our model’s performance could be measured against that of other classifiers with enhanced precision and validity. Furthermore, to validate the accuracy and reliability of the BEC-Pred model, we performed extensive computational experiments by challenging the model to annotate the EC numbers for a dataset of uncharacterized reactions catalyzed by lipase and hydrolase that were additionally collected from literature, followed by validation analysis. BEC-Pred performed well in each of these tasks and provided accurate prediction of both Novozym 435 induced hydrolysis of BuDLa and BuLLa substrates and lipase-catalyzed single-step synthesis of 4-OI. These evidences indicated that BEC-Pred was able to directly mark the enzymatic reactions verified by in vitro experiments with excellent prediction performance. In summary, this model integrates multifaceted data related to the substrates and products of biocatalytic reactions, enabling it to predict enzyme-catalyzed reactions of all categories with optimal accuracy among various machine learning methods. It also holds potential as an enzyme allocation module for enzyme-catalyzed biosynthesis planning algorithms [20]. Additionally, we have created reaction fingerprints from the learned representations, transforming sequences of enzyme-catalyzed reactions into vectors. Notably, BEC-Pred excels in predicting completely novel reactions not previously encountered in the dataset and shows promise in identifying diverse potential catalytic enzymes for biocatalytic organic reactions. This advancement positions our model as an invaluable tool for chemists and biologists, facilitating the systematic exploration of the enzymatic reaction landscape.

## Methods

### Datasets

We used a labelled set of chemical reactions to train the Transformer-based deep learning model [16]. Particularly, Probst et al. curated a data set, named ECREACT [21]. By combining the data of BRENDA, PathBank, Rhea and MetaNetX [22–25], enzyme-catalyzed reaction records were screened and sorted, and the corresponding EC number of each reaction was determined. Further processing of this dataset was carried out by eliminating products that occur as reactants in the same reaction, the removal of known co-enzymes, common by-products, the reaction with more than one product or no reactants (missing data) and so on [21]. Following this, for further preprocessing, the ECREACT dataset was first separated into two parts: the 'substrate-product' reaction parts in SMILES (Simplified Molecular Input Line Entry System) format and the EC number tags. We then refined the dataset by removing reactions that only had EC-level 1 classification. To better organize the data, we labeled the EC numbers associated with the reaction SMILES from EC-level 3, resulting in a total of 308 labels. The final dataset contains 56,512 enzymatic reactions with 308 EC sub-subclass labels. The final dataset was split five times using different random seeds, adhering to an 8:1:1 ratio for creating separate training, validation, and test sets. It is critical to note that all models developed in this study shared the same dataset splits for training, validation, and testing, ensuring uniformity and comparability across the constructed models.

The chemical reaction dataset USPTO used for pre-training model was made available by Lowe et al. [26]. The data consisted of 1.1 million reactions derived from the US patent reactions. A careful verification of the data sets showed that the overlap between the USPTO and ECREACT was only 0.00015%, and that the reaction class labels between the two datasets had different meanings, so concerns about potential data leakage during the subsequent fine-tuning phase of model training were almost negligible.

### Transfer learning

One approach to conduct transfer learning is to leverage a pretrained model that has learned basic representations that are relevant for the downstream task. With proper pre-training, a model can more easily focus on fine-grained details pertaining to the downstream task during the fine-tuned stage. Transfer learning has been successfully applied to a wide range of computer vision tasks, such as image classification and multilingual text classification [27–29]. This popular deep-learning technique has also been attempted in the field of organic chemistry, particularly in the field of synthesis planning and reaction classification [15, 30]. In this project, since the amount of experimental data on enzymatic reactions is not sufficient to train a sophisticated BERT model from scratch, we adopted the USPTO dataset, which comprises over 1.1 million organic chemical reactions, for pre-training the model weights. The USPTO dataset, rich in diverse organic reactions, serves as an ideal foundation for the model to learn a broad spectrum of chemical knowledge and SMILES syntax. This pre-training process enables the model to establish a robust baseline understanding of chemical reactions, which is essential for interpreting the more specialized data of enzyme-catalyzed reactions.

Subsequently, the model undergoes fine-tuning on the ECREACT dataset, a curated collection of enzyme-catalyzed reactions. This fine-tuning phase is critical as it adjusts the model’s learned representations from the USPTO dataset to the specific nuances and complexities of enzymatic reactions. By applying the knowledge gained from the USPTO dataset to the ECREACT dataset, the model learns to discern the unique characteristics of enzyme-catalyzed reactions. This transfer learning strategy not only compensates for the scarcity of enzymatic reaction data but also equips the model with the capability to generalize across a wide range of reaction types, thus handling the diversity and complexity inherent in enzyme-catalyzed reactions more effectively.

### Model architecture and baseline methods

**Fig. 1:**
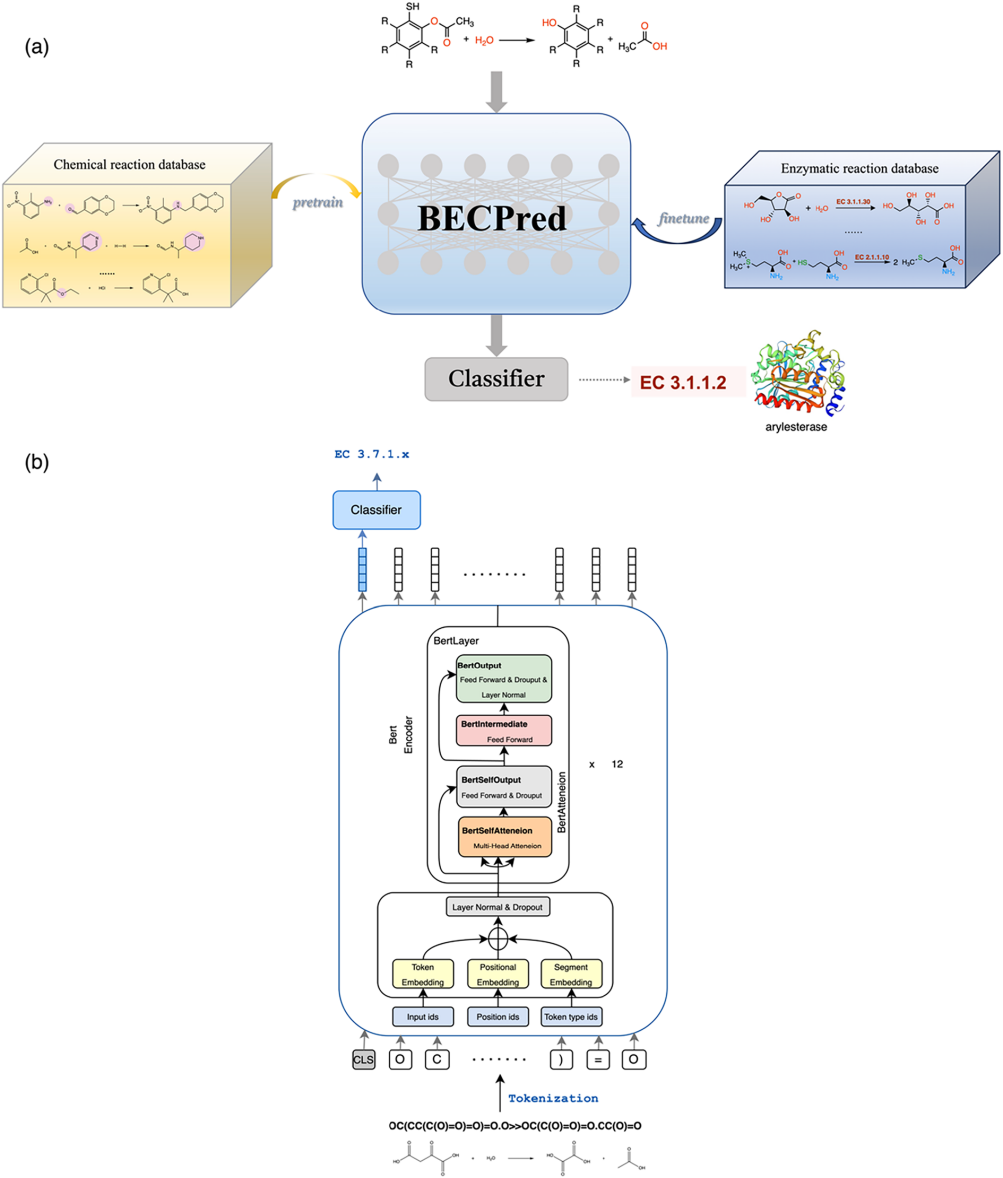
Overview of model design and BERT-based classifier architecture. **a** The figure illustrates the step-by-step process of the model design pipeline. It involves a two-phase approach starting with pretraining, followed by fine-tuning on downstream tasks. **b** The overall architecture of the BERT classifier. The Bert model with multiple stacks of self-attention layers, each of which comprises several attention heads. The model was utilized for a chemical reaction classification task through a classifier head. Additionally, the encoding of the [CLS] token serves as the reaction learnable fingerprint

In this paper, we pre-train BERT model based on mask language model on 1.1 million USPTO organic chemical reactions, and the specific structure of the model is shown in Fig. 1b. Pretraining the model with organic reaction data facilitates the acquisition of chemical reaction rules, which are also applicable to enzymatic reactions. Subsequently, fine-tuning the pre-trained BERT model with an enzymatic reaction dataset ECREACT enables it to discern the finer nuances inherent in the reaction rules specific to enzymes. Our BERT model was built on the Huggingface Transformers [16]. The objective of the model in masked language modelling was to predict individual tokens of the input sequences that were masked with a probability of 0.15. Similarly to BERT training, SMILE sequence was preceded by a specific category token [CLS]. In contrast to conventional BERT pre-training methodologies, our approach omits the Next Sentence Prediction (NSP) task. Additionally, we have further refined the pre-trained model by integrating a classifier head to facilitate multi-class enzymatic reaction classification. The [CLS] token embedding was used as an input to the classifier head. The output of the model was a probability value indicating the likelihood of each sample belonging to a certain category label. For fine-tuning, we employed the Adam optimizer for the last output layer and preserved the weight of the pre-trained model to augment its classification abilities. The fine-tuned model, with a classifier header added to the original BERT structure, was designed to classify non-masked [CLS] tokens. Key hyperparameters for our BERT model included a hidden size and intermediate size of 512, attention heads set to 4, and a learning rate of $1\times {10}^{-5}$, while the rest of the parameters were maintained as suggested in ref. [31]. We maintained the maximum sequence length at 512 tokens and conducted the fine-tuning over 50 epochs.

In order to conduct a thorough and comprehensive comparison of the proposed BERT model with other established methods, we have referred to the previous work by Watanabe et al. [14]. More specifically, to enable an equitable comparison, we used the same enzymatic reaction dataset as the baseline for all the models. We then trained K-Nearest Neighbor (KNN) classifier, Random Forest (RF) classifier, and a 4-layer deep neural network (DNN) classification model based on chemical reaction differential fingerprinting. By taking this approach, we were able to ensure that our results could be fairly and directly compared to other methods.

Additionally, we evaluated KNN and 4-layer DNN classifiers on the differential reaction fingerprint DRFP [18]. The DRFP algorithm operates by taking a reaction SMILES as input and generating a binary fingerprint. This fingerprint is derived from the symmetric difference of two sets, each composed of circular molecular n-grams. These n-grams are extracted from the molecules positioned on the left and right sides of the reaction arrow, without the need to differentiate between reactants and reagents. Recent studies have demonstrated that DRFP provides state-of-the-art reaction representations, exemplified by reaction yield predictions. It performs comparably to, if not better than, DFT-derived descriptors or transformer-based methods in yield prediction tasks for organic reactions [18]. The results indicate that DRFP molecular fingerprints outperform other structure-based fingerprints in reaction classification. Consequently, we employed DRFP to characterize enzyme-catalyzed reactions and utilized various methods for classification prediction.

Moreover, we conducted an investigation into a classification method that utilizes molecular graph representations [19]. These reaction graphs, detailed representations of molecules, include key structural and compositional characteristics. They serve as inputs for a graph convolutional network (GCN), which is specifically designed for the task of reaction classification. This method involves converting molecular structures into graph formats, where atoms are represented as nodes, each encoded with distinct features like atomic symbol, formal charge, and hybridization state. Bonds, depicted as edges, connect these nodes and are characterized by bond types, such as single, double, or aromatic. Subsequently, the GCN processes these graphs, using convolutional layers to update and aggregate the feature representations of atoms to generate a molecular-level representation. After obtaining the molecular-level representation, the representation of the chemical reaction is derived by calculating the difference between the graph representations of the reaction’s products and substrates. This overall reaction representation is then used for the reaction classification task. This approach has demonstrated significant potential to advance the field of chemical reaction prediction research in recent years by yielding more accurate models [32–36]. These alternative models were selected to provide a broad range of comparative approaches against our proposed BERT model. This allowed us to achieve a thorough and meaningful assessment of our model's efficacy and to make informed conclusions regarding its performance relative to its counterparts.

### TMAP

TMAP [17] is an efficient way to reduce the size of the dataset. Relative to other dimension reducing methods, TMAP has the merit of having two-dimensional tree like output, which retains the local and global structures at the same time. This method includes four steps: (1) LSH Forest-based indexing; (2) generating k nearest-neighbor maps; (3) applying Kurskal's algorithm to compute minimal spanning tree; and (4) building a tree. The resulting layout is then shown with the Faerun [37] Interactive Data Visualization Framework. TMAP [17] and Faerun [37] were initially designed for visualization of big molecules, but they have proven useful for many other types of data. In this section, we use a custom edition of Smith Drawer, which is expanded to enable the presentation of chemistry.

### Assessment metrics

In order to compare the results with the CEN, we have computed the confusion entropy as shown below.

1 $${\text{ACC}}=\frac{{\text{TP}}+{\text{TN}}}{{\text{TP}}+{\text{FN}}+{\text{TN}}+{\text{FP}}}$$

2 $${\text{MCC}}=\frac{{\text{TP}}\times {\text{TN}}-{\text{FP}}\times {\text{FN}}}{\sqrt{\left({\text{TN}}+{\text{FN}}\right)\times \left({\text{TN}}+{\text{FP}}\right)\times \left({\text{TP}}+{\text{FN}}\right)\times \left({\text{TP}}+{\text{FP}}\right)}}$$

3 $${{\text{Precision}}}_{i}=\frac{{{\text{TP}}}_{i}}{{{\text{TP}}}_{i}+{{\text{FP}}}_{i}}$$

4 $${{\text{Precision}}}_{weighted}=\frac{{\sum }_{i=1}^{L}{({\text{precision}}}_{i}\times {\omega }_{i})}{\left|L\right|}$$

5 $${{\text{Recall}}}_{i}=\frac{{{\text{TP}}}_{i}}{{{\text{TP}}}_{i}+{{\text{FN}}}_{i}}$$

6 $${{\text{Recall}}}_{weighted}=\frac{{\sum }_{i=1}^{L}{({\text{Recall}}}_{i}\times {\omega }_{i})}{\left|L\right|}$$

7 $${{\text{F}}1}_{weighted}=\frac{2\times {{\text{Precision}}}_{weighted}\times {{\text{Recall}}}_{weighted}}{{{\text{Precision}}}_{weighted}+{{\text{Recall}}}_{weighted}}$$

where TP, TN, FP, and FN denote true positives, true negatives, false positives, and false negatives, respectively. TP and TN represent the number of samples that were correctly classified as positive and negative, respectively.

All of the above indicators are used to evaluate the performance of any of the classifiers mentioned in this study. Accuracy, Matthews correlation coefficient (MCC) and F1-score are the more important indicators [38]. Accuracy is used to measure the performance of the model, but due to the imbalance in the classification of enzymes in the data set used for training, the results will be biased in terms of accuracy. The Matthews correlation coefficient is also known as balanced accuracy, and it is also used as a performance measure for classification problems when the data set shows class imbalance. The F1 Score, as the harmonic mean of accuracy rate and recall rate, presents a holistic view that accounts for both precision and recall in a classification model. Acknowledging the dataset’s imbalance, we calculate a weighted F1-score. This nuanced approach addresses the limitations of the macro F1-score by incorporating considerations for sample imbalances. In the computation of Precision and Recall, each category’s values are multiplied by the proportion of that category in the total sample, providing a more nuanced understanding of model performance across different classes. Additional metrics such as accuracy and recall rate are also presented for readers' reference, offering a comprehensive evaluation framework.

## Results and discussion

### Dataset description

**Fig. 2:**
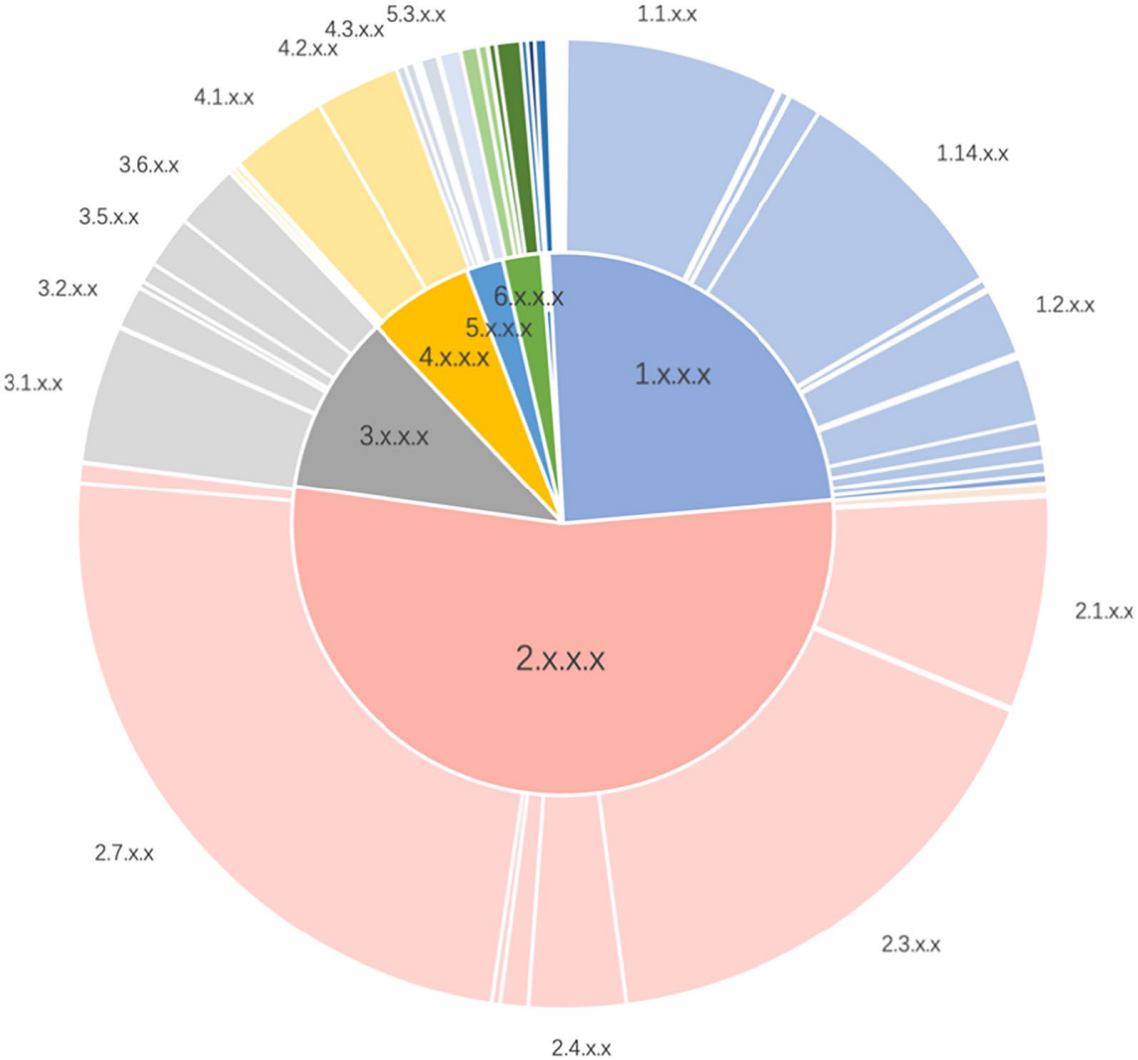
The distribution of samples at EC-levels 1 (corresponding to enzyme classes) and 2 (corresponding to enzyme sub-classes) for oxidoreductases (class 1), transferases (class 2), hydrolases (class 3), lyases (class 4), isomerases (class 5), ligases (class 6) and translocases (class 7), in the ECREACT dataset

The distribution of available data reveals a heavy imbalance in the distribution of enzyme-catalyzed reaction examples. At the EC-level 1, corresponding to enzyme classes, transferases (EC 2.x.x.x) represent 53% of the total entries, followed by oxidoreductases (EC 1.x.x.x) with 25%, hydrolases (EC 3.x.x.x) with 11%, lyases (EC 4.x.x.x) with 6%, the combined content of isomerases (EC 5.x.x.x), ligases (EC 6.x.x.x) and translocases (EC 7.x.x.x) with less than 5%. Among transferases, the most common subclasses at EC-level 2 are transferases transferring phosphorus-containing groups (EC 2.7.x.x) at 25%, and the details of the major subclasses included under the remaining EC classes are also shown in Fig. 2. Transferase-catalyzed reactions encompass few subclasses at EC-level 3 with large sample size, whereas oxidoreductase- and hydrolase-catalyzed reactions are divided into many subclasses with small sample size at EC-level 3. Lyases, isomerases, ligases, and translocases are split into fewer subclasses at EC-level 3, but most of them contain very few samples. Thus, to ensure a proper evaluation of data-driven models, the assessment of their performance must take into account the different populations of each subclass at EC-level 3. Furthermore, considering the sparse data in EC-level 4 classification presents a formidable challenge, as this level demands precise categorization of enzymatic reactions. The EC-level 4 classification necessitates comprehensive information regarding the chemical reactions enzymes catalyze, which are often highly diverse and complex. Sparse data complicates accurate classification of these reactions due to insufficient examples that encapsulate the entire spectrum of variability within each category. Consequently, our study focuses on predicting the first three digits of the EC number (EC sub-subclass). We organized the EC numbers associated with the reaction SMILES from EC-level 3, labeled them accordingly, and identified a total of 308 labels.

### Reaction classification

In this study, the ground truth data comprises chemical transformations presented in SMILES text format. To explore the expressive capabilities of reaction string embedding, we leveraged the BERT-based [31] model, initially pretrained on the USPTO dataset of chemical reactions [26].

To gauge the reliability of our model and compare it to the other baseline models, we used the metrics of accuracy, overall Matthews Correlation Coefficient (MCC), and Macro F1 Score, all of which are defined in terms of true positive (TP), true negative (TN), false negative (FN), and false positive (FP).

The results of enzyme catalyzed reactions dataset can be seen in Table 1. Experiments show that the method proposed in this paper can classify various categories with high accuracy.

**Table 1:** Predicted results at different EC-levels on the ECREACT test set

Data	ACC	MCC	F1-score
Level 1	$0.962\pm 0.003$	$0.945\pm 0.004$	$0.963\pm 0.003$
Level 2	$0.934\pm 0.003$	$0.928\pm 0.003$	$0.933\pm 0.003$
Level 3	$0.916\pm 0.003$	$0.907\pm 0.003$	$0.913\pm 0.004$

At the 1st-level, the Bert classifier model for enzymatic reactions demonstrated an accuracy of 96.2% on the ECREACT test set, with the overall Matthews correlation coefficient (MCC) at 94.5% and a weighted F1-score of 96.3%. At the 3rd-level, the classification model continued to show robust performance with an accuracy of 91.6%, an MCC of 90.7%, and a macro F1-score of 91.3% on the test set.

**Fig. 3:**
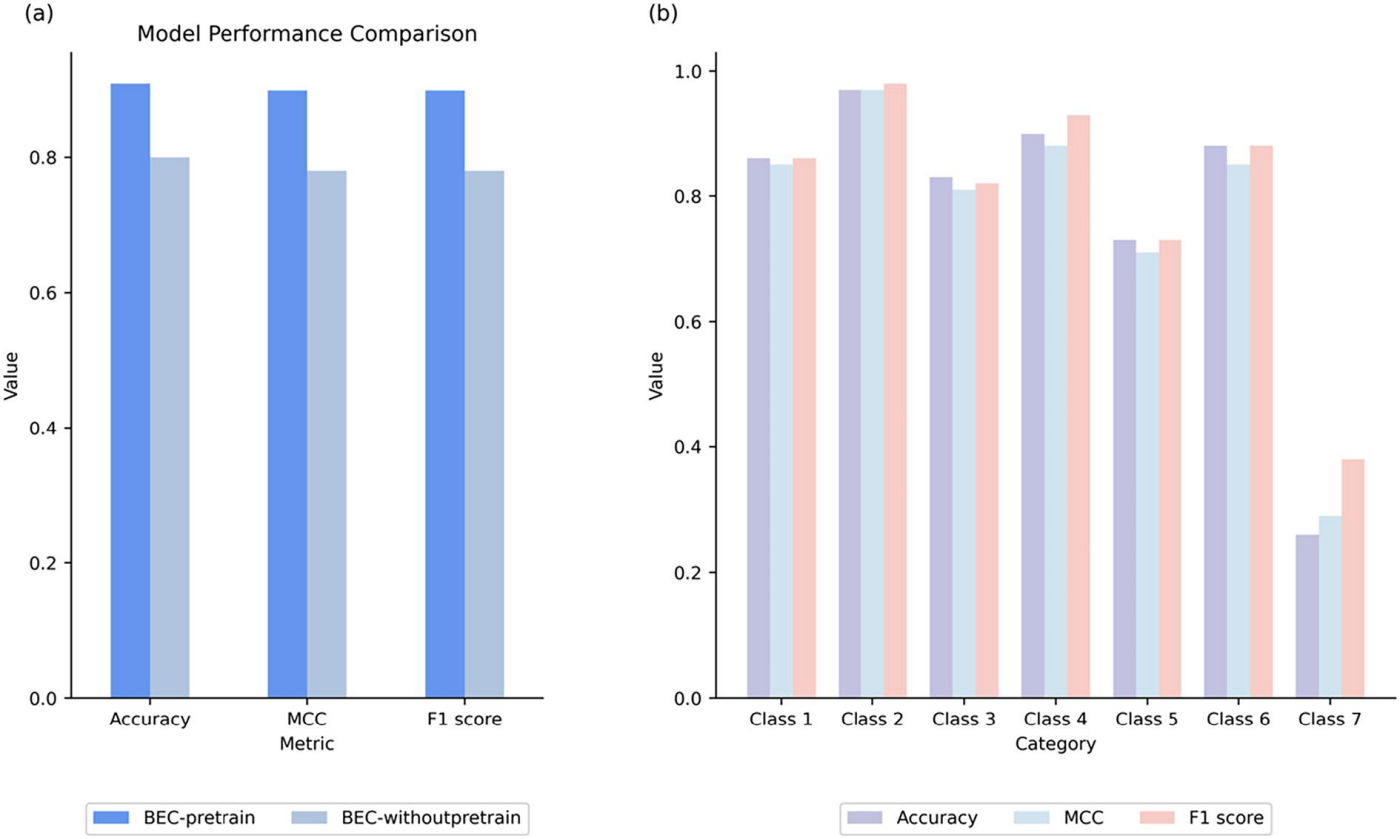
**a** The figure illustrates a comparison between BERT classification results with and without pre-training on USPTO datasets. It is evident that the pre-trained BERT model consistently outperforms the non-pre-trained model across all evaluation metrics; **b** The figure below displays the prediction results of BEC-Pred for 7 categories in the test set. Different colors represent the three evaluation indicators. Remarkably, the bc-pred model achieved the highest accuracy for categories 1, 2, 4 and 6 among all seven categories. Moreover, the model demonstrated moderate accuracy for categories 3 and 5, while the category 7 exhibited relatively lower accuracy

Additionally, our model predicted EC-level 3 tokens for seven classes of enzymes, as shown in Fig. 3b. The analysis of the model demonstrated an excellent performance of transferase-catalyzed reactions (class 2), achieving a 99% accuracy. However, the accuracy of its predictions for oxidative reductases (class 1) is relatively low due to the presence of numerous EC-level 3 subclasses. Each subclass, recorded in the database, comprises only a small sample size, resulting in a higher diversity of substrates and products compared to other classes. Notably, the prediction of enzymes (EC number) exhibited the lowest accuracy when predicting translocases. This discrepancy can be attributed to the imbalance in the training data.

This result confirms that although the model can distinguish between different categories of enzyme-catalyzed reactions based on the given dataset, it fails to achieve satisfactory prediction accuracy in specific categories due to insufficient training data. The distribution of data shows a significant imbalance in the substrate-product samples. In EC-level 3, a few subcategories of transferase reactions (EC Class 2) have a large sample size. Conversely, reactions catalyzed by oxidoreductases (EC Class 1) and hydrolases (EC Class 3) are classified into numerous sub-subclasses. Many of these sub-subclasses comprise a limited number of samples, posing challenges for comprehensive analysis and accurate prediction. The observed lower overall accuracy in isomerases (EC Class 5) primarily results from their unique function of catalyzing intramolecular modifications. These modifications might be mistakenly predicted as being catalyzed by other enzymes if they were to occur intermolecularly. Additionally, the wide variety of these reactions further complicates their classification. On the other hand, reactions catalyzed by lyases (EC Class 4), ligases (EC Class 6) constitute a smaller proportion in EC-level 3 classification, although most of these subcategories contain only a few samples. Therefore, it is crucial to consider the varied populations within each sub-subclass and the diversity of reactions within a single class when assessing sub-subclasses at EC-level 3, to ensure accurate and appropriate evaluation.

### Comparison with other benchmark methods

To evaluate the performance of our proposed model in comparison to the current leading solutions in machine learning-based chemical reaction prediction, we constructed six additional models for benchmarking. These included three models based on reaction differential fingerprint, two models based on drfp fingerprint embedding, and one gnn classification model. When compared to our model using the identical test set, this study observed that BEC-Pred model achieved the highest accuracy, MCC, and F1 score among the models utilizing enzyme reaction embedding.

Table 2 provides a profound comparison between BEC-Pred and other traditional classification methods such as KNN, RF, MLP, and GNN. BEC-Pred is a Bert model that has been pretrained extensively on the USPTO reaction dataset and fine-tuned according to specific tasks. It boasts the highest accuracy in the validation set, at 0.916, with a Matthews correlation coefficient (MCC) of 0.907 and an F1 score of 0.913. These results are significantly superior to other models, including KNN-morgan2, RF-morgan2, MLP-morgan2, KNN-drfp, MLP-drfp, and Graph Convolutional Network (GCN). Models like KNN-morgan2 and MLP-morgan2 also exhibit good accuracy and F1 scores, but BEC-Pred still outperforms them. The RF-morgan2 model has the lowest F1 score of 0.739, indicating the lowest precision and recall among all models. Overall, our BEC-Pred model surpasses all other models in terms of accuracy, MCC, and F1 scores, making it the best choice for the given task. This pretrained Bert-based model demonstrates the powerful capabilities of transformer architecture by leveraging their ability to capture deep contextual relationships within data, a significant advancement beyond the capabilities of models like KNN, RF, MLP, and GNN. While these traditional models are useful, they cannot match the complex pattern recognition and feature extraction abilities of BEC-Pred. Particularly, BEC-Pred’s advanced understanding of sequences and context greatly enhances the accuracy of predictions, making it a valuable tool for tasks requiring nuanced differentiation. Furthermore, the model's pre-training allows it to assimilate a vast array of unlabeled data from the USPTO reaction datasets, thereby augmenting its generalization capabilities and making it exceptionally proficient in fine-tuning across varied downstream datasets—an evident superiority over traditional machine learning alternatives.

**Table 2:** Comparison of the predictive performance of our BEC-Pred model with other machine learning methods at level 3 of the EC number

Data	ACC	MCC	F1 score
KNN-morgan2	$0.843\pm 0.005$	$0.827\pm 0.005$	$0.843\pm 0.005$
RF-morgan2	$0.768\pm 0.003$	$0.744\pm 0.005$	$0.739\pm 0.005$
DNN—morgan2	$0.861\pm 0.005$	$0.847\pm 0.005$	$0.848\pm 0.006$
KNN-drfp	$0.837\pm 0.006$	$0.820\pm 0.005$	$0.837\pm 0.006$
DNN-drfp	$0.852\pm 0.004$	$0.834\pm 0.005$	$0.837\pm 0.004$
GNN	$0.861\pm 0.002$	$0.847\pm 0.002$	$0.854\pm 0.003$
BEC-Pred (ours)	$0.916\pm 0.003$	$0.907\pm 0.003$	$0.913\pm 0.004$

The evaluation criteria used include accuracy, MCC, and F1 scores

To validate the effectiveness of our pre-training strategy, we conducted a comparative performance analysis by training the BERT model on the ECREACT [21] dataset from scratch, using the same parameters as the fine-tuned model. The outcomes of this experiment, demonstrating the impact of the pre-training approach, are presented in Fig. 3a. The results indicate that the BERT model, once pre-trained, outperforms its unpre-trained counterpart with a significant margin in terms of classification accuracy. This suggests that the transfer learning process effectively internalizes the intrinsic patterns and relationships characteristic of chemical reactions present in the USPTO datasets. Furthermore, it demonstrates the model’s capability to efficiently transfer the acquired knowledge to subsequent tasks involving the classification of reactions.

**Fig. 4:**
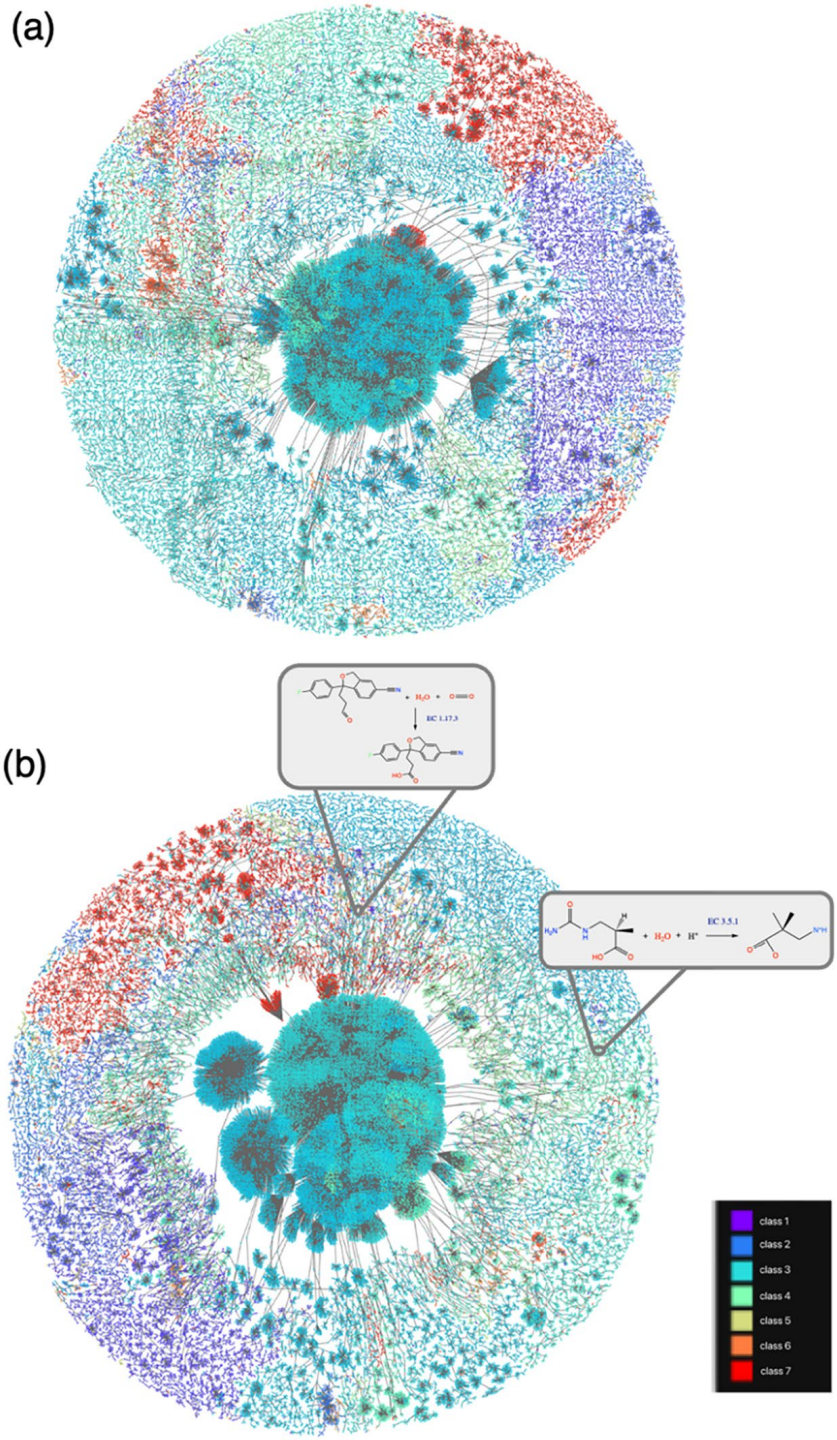
Response Atlas. Specifically, **a** and **b** present visualizations of reaction fingerprints generated by the unpre-trained and pre-trained BERT models, respectively, using the ECREACT dataset

To demonstrate that the impressive performance of BEC-Pred, as summarized in Table 2, indicates its ability to learn highly relevant chemical rules for EC classification, we employed the TMAP [17] and Faerun [37] visualization library to visualize the reaction fingerprints of enzyme-catalyzed reactions embedded in pre-trained and unpre-trained models(Fig. 4); each label is denoted by a distinct color.

Molecular fingerprinting is a widely used method for analyzing molecules with similar structures and chemical compositions [39–42]. In our study, the [CLS] token—consistently unmasked throughout both pre-training and fine-tuning phases—serves as a BERT fingerprint, embedding a global descriptor of the reaction. This constant engagement allows the model to effectively recover any tokens that have been masked within the sequence. Specifically, the embedding of the [CLS] token in our model is a 512-dimensional vector, aligning with the hidden layer size of the standard BERT model.

As depicted in Fig. 4, the rainbow colors and their shades represent 308 enzymatic reaction labels, providing critical insights into the spatial distribution of enzymatic reactions at the molecular level and substantiates the effective clustering of diverse enzymatic reactions. Notably, the reaction clusters within the pre-trained model (Fig. 4b) exhibit a tighter association than those in the non-pre-trained model (Fig. 4a). Specifically, Fig. 4b demonstrates the pre-trained BERT model’s enhanced ability to differentiate between hydrolase enzymes (EC 3.x.x), thereby substantiating the pre-trained model's superior performance in categorizing related reaction classes. In Fig. 4b, we also present the detailed view of this reaction diagram, demonstrating that through visual inspection of the TMAP, reactions catalyzed by the same enzyme can be easily identified in proximity to the given reaction. This comparison confirms that the fine-tuned BEC-Pred model excels in the classification of enzyme-catalyzed reactions, with generated reaction fingerprints capable of distinguishing various chemical components and effectively differentiating types of enzymatic catalysis.

### Use-cases of classification prediction models

To evaluate the efficacy of BEC-Pred in addressing the challenges of predicting actual enzyme-catalyzed reactions, we conducted predictive validations for reactions catalyzed by lipases and hydrolases. This involved assessing the potential for practical application of the enzyme reaction classification prediction model by juxtaposing enzyme classification data from actual experiments with model predictions. Lipases and hydratases are particularly suited for such research due to their broad substrate spectra, coupled with their high substrate, regio-, and enantioselectivity [43, 44]. These enzymes have garnered extensive research interest and data support within academic circles. There is a wealth of experimental data available, encompassing their catalytic mechanisms, structures, and properties, making them ideal subjects for investigation.

Lipase (EC 3.1.1.3) is a widely distributed protease belonging to the α/β serine hydrolase family, which exists in vivo [45, 46] The important role of natural lipase in nature is the hydrolysis of triglyceride (TAG) and the formation of free fatty acid (FFA).

Water molecules are selectively added to their primary, secondary, or tertiary alcohols in the FFA carbon–carbon double bond [47]. Hydrase is a highly efficient asymmetric catalytic system, which has important application value in organic synthesis [47]. Among the current hydratases, keto hydratase, catalpa lime hydratase and acetylene hydratase are considered to be the most important hydratase, which can perform enzyme addition of water hydratase and free fatty acids.

Therefore, our model BEC-Pred selected an additional set of 24 lipase- and hydratase-catalyzed reactions (not included in the training dataset) from BRENDA [25] and SABIO-RK [48], another biochemical reaction database, to serve as an external test set for model validation. Remarkably, on this extra test set, BEC-Pred achieved a prediction accuracy of 83.3% and a Matthew’s Correlation Coefficient (MCC) of 73.5%, underscoring its robust predictive capability. Table 3 meticulously outlines both sets of successful and unsuccessful predictions, alongside their respective ground truths. Successful example 1–9 reflect the ability of the model to predict the enzymes required for novel enzymatic reactions.

**Table 3:** Predict application instances. For each response, basic facts are shown in black, and predictions that are wrong are shown in red

No	Rxn SMILES	True/predicted
1		3.1.1/3.1.1
2		3.1.1/3.1.1
3		3.1.1/3.1.1
4		3.1.1/3.1.1
5		4.2.1/4.2.1
6		4.2.1/4.2.1
7		4.2.1/4.2.1
8		4.2.1/4.2.1
9		4.2.1/4.2.1
10		4.2.1/1.1.2
11		3.1.1/3.7.1

In the unsuccessful example (10), the model failed to predict enzymatic reactions to hydrolysis of 2-hydroxysuccinic acid. The reaction was incorrectly identified as one catalyzed by an enzyme classified under EC 1.1.2.x, which typically involves the dehydrogenation of alcohols and the concomitant transfer of hydrogen ions. The confusion may arise because the hydrolysis of 2-hydroxysuccinic acid, while not directly a dehydrogenation reaction, does involve the removal of hydrogen atoms as part of the water molecule released. This subtle but critical distinction between hydrolysis and dehydrogenation processes might have led the model to erroneously associate the reaction with cytochrome-dependent enzymes that operate under the 1.1.2.x classification. Example (11) highlighted the prediction of different EC-level 3 tokens to catalyze the reaction. Given the ground truth that the reaction is catalyzed by the Carboxylic-ester hydrolase (3.1.1.x), the prediction suggests that the reaction is catalyzed by an in ketonic substance (3.7.1.x). This choice likely reflects a potential bias influenced by the uneven number of training samples in the two sub-subclasses, with 979 reactions categorized under EC 3.1.1 and only 61 under EC 3.7.1.

**Fig. 5:**
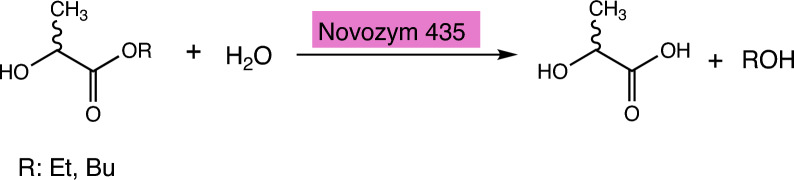
Novozym 435 induced hydrolysis of BuDLa and BuLLa substrates

Lipase, as a biocatalyte, has shown satisfactory catalytic ability in hydroxyaldehyde condensation, Michael addition, Knoevenagel condensation and other reactions. It has been reviewed that lipase as a catalyst can induce various polycondensation reactions to produce various new polyester [49]. Novozym 435 (EC 3.1.1.3), as a biological catalyst, can induce the hydrolysis of BuDLa and BuLLa substrates [50]. In the absence of this enzyme, no hydrolysis reaction occurs under similar reaction conditions.This reaction can be successfully predicted as EC 3.1.1 by our model, with the reaction pathway illustrated in Fig. 5.

Monooctyl 4-iconate (4-OI), a cell permeable lipid derivative of iconate, has shown great medical potential in treating multiple sclerosis, tinea leucoides, lupus erythematosus and other diseases by means of immune regulation. In addition, in the novel coronavirus pneumonia and a variety of human pathogenic virus infection has shown strong antiviral and anti-inflammatory dual effects.

**Fig. 6:**
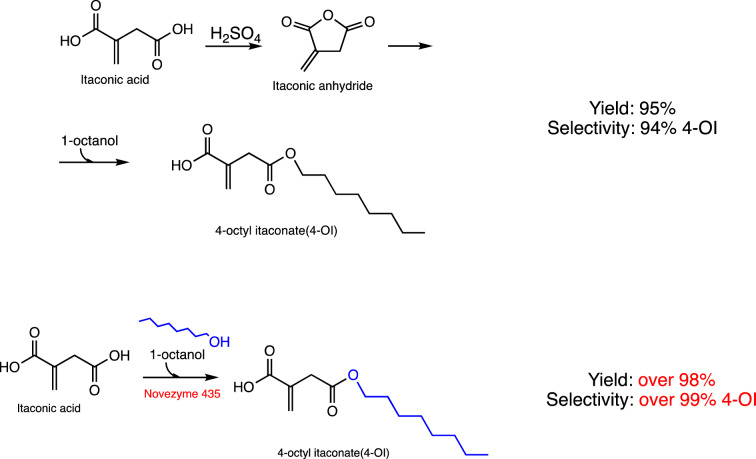
Synthesis Route of 4-OI

The two-step synthesis route for 4-OI, as previously reported, exhibited a high selectivity (94%) and yield (95%). However, the synthesis involves significant storage costs for the intermediate, Itaconic Anhydride. Additionally, the production of Itaconic Anhydride necessitates the use of acidic catalysts and high-temperature conditions, coupled with extensive purification processes. These requirements not only increase the potential for environmental pollution but also escalate production costs. Liu et al. [51] investigated an innovative green synthetic method, efficiently producing 4-OI in a single step. This process utilizes lipase (EC 3.1.1.3) catalysis, employing Itaconic Acid (IA) and Octanol. Their research revealed that Novozym 435 (CALB)’s distinctive cavity structure, in conjunction with the solvent shrinkage effect, facilitates an almost complete direct conversion of IA and Octanol to 4-OI (nearly 100% efficiency). This method is in line with green engineering principles, aiming to minimize operational steps and reduce energy consumption. However, the current chemical synthesis route of 4-OI has some shortcomings, such as low selectivity, low yield, complicated process, serious pollution and high price. Liu et al. [51] explored a new green process for the efficient synthesis of 4-OI from iconic acid (IA) and octanol in one step catalyzed by lipase (EC 3.1.1.3). Different methods for the synthesis route of 4-OI are illustrated in Fig. 6. BEC-Pred can successfully predict the method that Carboxylic-ester hydrolases (EC 3.1.1) can catalyze the one-step synthesis of 4-OI between IA and octanol, and BEC-Pred can also successfully predict the EC number. This result also demonstrates that BEC-Pred holds great potential for use in the chemical synthesis industry.

## Conclusion

In this study, we developed BEC-Pred, a molecular converter-based multi-classification model, for predicting Enzyme Committee (EC) numbers. Our model demonstrates significant improvement in overall precision compared to other machine learning methods, evidenced by an accuracy of 91.6%, MCC of 90.7%, and an F1 score of 91.3%. The unique Bert fingerprint generated by BEC-Pred offers an in-depth characterization of enzyme-catalyzed reactions, contributing to a better understanding of enzymatic behavior and catalysis. This advancement holds promise for innovative applications in chemical biology, synthetic biology, and drug metabolism. To further illustrate the usefulness of BEC-Pred beyond simple benchmarks, we discussed several catalytic examples of lipases and hydrolases as verified by BEC-Pred model, and successful prediction of Novozym 435-induced hydrolysis of BuDLa and BuLLa substrates and lipase-catalyzed single-step synthesis of 4-OI, which were useful real-wrold cases collected from literatures. These examples demonstrated that BEC-Pred model can retrieve reaction metadata, and provide useful assistance in exploring biocataltic processes. The latest iteration of BEC-Pred demonstrates significant predictive accuracy, yet it faces challenges. The dataset's imbalance notably affects the precision in categorizing EC sub-subclasses within enzymatic reactions, particularly for those with limited sample representation. Enhancing the model’s performance necessitates not only dataset expansion but also the incorporation of more comprehensive experimental data. This would substantially improve the model's predictive prowess. Looking ahead, BEC-Pred possesses vast potential for wider applications. It has the capability to revolutionize enzyme screening for specific reactions, enhance enzymatic reaction identification accuracy, assist in predicting enzyme functions in less-explored species, and provide a vital enzyme allocation function in the algorithm for biosynthesis planning. Furthermore, the seamless integration of CLEAN and BEC-Pred enables the efficient screening of enzymes for specific chemical reactions, offering a reliable reference for classifying a broader range of unannotated catalytic reactions. Such progress could be groundbreaking in enzyme engineering, drug discovery, and the synthesis of novel biomolecules, where a detailed understanding of enzyme functions is crucial.

## Data Availability

The trained models and code are publicly available at https://github.com/KeeliaQWJ/BEC-Pred. The raw ECREACT dataset is available at the URL https://github.com/rxn4chemistry/biocatalysis-model.
